# Potential Risk Factors for Isolated Hypothyroxinemia in Women of Childbearing Age—Results from Retrospective Analysis

**DOI:** 10.3390/jcm10225384

**Published:** 2021-11-18

**Authors:** Małgorzata Karbownik-Lewińska, Jan Stępniak, Andrzej Lewiński

**Affiliations:** 1 Department of Oncological Endocrinology, Medical University of Lodz, 90-752 Lodz, Poland; jan.stepniak@umed.lodz.pl; 2 Department of Endocrinology and Metabolic Diseases, Polish Mother’s Memorial Hospital-Research Institute, 93-338 Lodz, Poland; andrzej.lewinski@umed.lodz.pl; 3 Department of Endocrinology and Metabolic Diseases, Medical University of Lodz, 93-338 Lodz, Poland

**Keywords:** isolated hypothyroxinemia, childbearing age, vitamin D deficiency, insulin resistance, reproduction

## Abstract

Isolated hypothyroxinemia (IH) unfavorably affects reproduction. This study aimed to evaluate retrospectively if any routinely measured clinical/laboratory parameters are associated with IH among women of childbearing age hospitalized in the endocrine department. A group of 466 female non-pregnant inpatients (age range 13–57 years) was considered. IH (decreased free thyroxine (FT4) with normal TSH) was found in 8/466 patients (1.72%). Vitamin D deficiency (<30 ng/mL) was found in all patients with IH, whereas severe Vitamin D deficiency (<20 ng/mL) was found in 5/6. Vitamin D concentration was lower in IH females. FT4 concentration was lower in patients with severe vitamin D deficiency and correlated positively with vitamin D concentration. Insulin resistance index (IRI) was increased (>1.25) in 5/6 patients with IH. IRI was higher in IH patients and it was the only independent linear factor for IH in the univariate regression. FT4 concentration was lower in patients with increased IRI and correlated negatively with IRI. FT4 concentration correlated negatively with body mass index (BMI) and LDL cholesterol or triglycerides, and positively with HDL cholesterol or HDLC/cholesterol ratio. Vitamin D deficiency, insulin resistance and increased BMI (as potential causative factors), and abnormal lipid profile (as a possible consequence), are associated with IH in women of childbearing age. Eliminating risk factors for hypothyroxinemia may improve reproductive health.

## 1. Introduction

Isolated hypothyroxinemia (IH) during pregnancy is defined as a decreased of free thyroxine (FT4) concentration below the 2.5th percentile (however different FT4 percentile cutoffs are used) with a normal TSH [1]. The prevalence of IH in the pregnant population ranges from 1.3% to 23.9% depending on the study [2], with the most frequently reported percentage being 8–10% [3,4]. In some studies it was found to be even more common than subclinical hypothyroidism [4]. A recently published meta-analysis revealed that the pooled prevalence rate for IH during pregnancy (calculated from 63 studies, taking into account TSH cutoff and timing of screening) is 2.05% [5].

Isolated hypothyroxinemia in pregnancy is associated with unfavorable pregnancy outcomes, such as preterm delivery [6,7,8], preterm premature rupture of the membranes [9], spontaneous abortion [10], gestational diabetes [11], gestational hypertension [12], preeclampsia [13], and low birth weight [9], and also high birth weight [14] or macrosomia [15], as well as impaired neuropsychological development of the offspring [16]. The prevalence of IH was found to be higher in women with gestational diabetes mellitus in a moderately iodine-deficient area [17], and the association between low FT4 concentration and gestational diabetes mellitus in the first and the second trimester of pregnancy has been confirmed in a recently published meta-analysis [11]. Interestingly, FT4 concentration in cord blood was found to be higher in the low-risk pregnant patients compared to high-risk individuals [18]. The relationship between IH and adverse pregnancy outcomes has been recently summarized [19]. Preterm birth is the unfavorable effect of hypothyroxinemia that is most frequently documented in the literature [19]. However, there are also published data available that do not show increased incidence rates of adverse maternal outcomes and perinatal complications in patients with IH in the first two trimesters [20].

Although an undesired contribution of IH to reproduction is not confirmed in all studies, its potential harmful effects should not be neglected with respect to gestation and the preconception period. Unfortunately, optimal strategies in the case of borderline states of thyroid hypofunction, i.e., subclinical hypothyroidism and IH, have not been precisely formulated due to the lack of controlled interventional trials [1,21]. However, we should still try to optimize the management of both abnormalities in pregnant women and at preconception. Regarding IH, at least two limitations occur, making this issue more difficult. The first one is that screening tests at preconception and during pregnancy do not comprise thyroxine concentration measurement [1], making a diagnosis impossible in all individuals with IH, and the second one is that statements of whether to treat or not treat pregnant patients with this kind of thyroid hypofunction are not univocal [1,22,23,24], and regarding women planning pregnancy, this issue is not even discussed in the literature.

On the other hand, at the step when the final diagnosis of either subclinical hypothyroidism or IH is formulated, the latter gives more tools to prevent this mild thyroid hypofunction. Whereas subclinical hypothyroidism is usually associated with autoimmune process and, therefore, it is not preventable, to identify risk factors for IH allows one to eliminate them and, simultaneously, to prevent direct adverse effects of these factors on reproduction and general health.

Until now, six main risk factors of IH are accepted in pregnant women, i.e., iodine deficiency, iron deficiency, obesity, older age, environmental disruptors, and an imbalance between pro- and antiangiogenic factors [2,23]. Regarding the non-pregnant population of childbearing age, the same risk factors are expected, however they have not been previously discussed in the literature.

The aim of this study was to check if any routinely measured clinical or laboratory parameters are associated with isolated hypothyroxinemia among women of childbearing age hospitalized in an endocrine department, i.e., a group of individuals not representative of the general population.

## 2. Materials and Methods

Ethical approval of the study was obtained from the Ethical Committee of the Polish Mother’s Memorial Hospital-Research Institute, Lodz, Poland (No. 40/2018).

The steps of this retrospective study, including the patient characteristics, were described in detail elsewhere [25].

The following clinical/laboratory parameters were taken into account in statistical evaluation: age, body mass, height, body mass index (BMI), red blood cells (RBC), hemoglobin (Hgb), white blood cells (WBC), neutrophils, lymphocytes, platelets, total cholesterol, HDL cholesterol (HDLC), LDL cholesterol (LDLC), HDLC/cholesterol ratio, triglycerides (TGs), glucose, iron concentration, vitamin D, insulin resistance index (IRI), and thyroid tests, including thyroid antibodies. IRI was calculated on the basis of glucose and insulin concentrations obtained during an oral glucose tolerance test (OGTT) [26].

Thyroid tests and their reference ranges were as follows: thyrotropin (TSH; 0.27–4.2 mIU/L), free thyroxine (FT4; 0.93–1.7 ng/dL) and free triiodothyronine (FT3; 2.6–4.4 pg/mL), and thyroid antibodies, i.e., thyroid peroxidase antibodies (TPOAb; <34 IU/mL), thyroglobulin antibodies (TgAb; <115 IU/mL), and TSH receptor antibodies (TSHRAb; <1.75 IU/mL).

Reference values for FT4 (immunochemiluminescent test) correspond to the 2.5th and 97.5th percentile of results from healthy test subjects. Therefore, isolated hypothyroxinemia was defined in the present study as a FT4 concentration below the 2.5th percentile.

From the whole group of 466 female inpatients (age range 13–57 years), the following groups were selected to evaluate differences between the examined group (i.e., patients with isolated hypothyroxinemia) and any other chosen group, and to evaluate the frequency of events:

- patients with isolated hypothyroxinemia (IH; decreased FT4 and normal TSH and FT3), *n* = 8.
- patients with normal thyroid tests (TSH, FT3 and FT4 in reference ranges; comprising also patients on L-thyroxine treatment), *n* = 280.
- patients with normal thyroid tests after excluding individuals on L-thyroxine treatment (normal thyroid function without any treatment), *n* = 240.
- the whole group of 466 patients minus patients with IH, *n* = 458.
- the whole group of 466 patients minus patients with IH after excluding individuals on L-thyroxine treatment, *n* = 352.

Additionally, the following groups were considered to evaluate correlations between linear parameters and to perform regression analysis:

- patients with normal TSH (comprising patients with IH and with normal thyroid tests), *n* = 288.
- patients with normal TSH after excluding individuals on L-thyroxine treatment, *n* = 248.
- the whole group of 466 patients.
- the whole group of 466 patients after excluding individuals on L-thyroxine treatment, *n* = 360.

Because ferritin concentration was measured in 23 out of 466 patients and was not recorded in any patient with IH, it was not considered in the statistical evaluation. Iron concentration was recorded in only 71 out of 466 patients, but it was not recorded in any patient with IH, therefore it was taken into account only in a part of the statistical evaluation. 

### Statistical Analyses

The data were statistically analyzed using following tests. The Student’s unpaired *t*-test was applied to compare differences between independent groups; the results are presented as means ± SEM. To evaluate the frequency of events we used the two-sided ratio comparison test. To evaluate possible correlations between two chosen linear parameters we used the Pearson’s correlation coefficient. To determine which continuous variable might have been associated with IH (FT4 < 0.93 ng/dL), we applied univariate logistic regression analysis. To compare the median between two groups we used the Mann–Whitney rank sum test. Statistical significance was determined at the level of *p* < 0.05. Multiple comparisons were corrected by false discovery rate (FDR) at the level of 0.1, which was achieved by the Benjamini–Hochberg procedure.

## 3. Results

Isolated hypothyroxinemia was found in 8 out of 466 female non-pregnant inpatients (1.72%).

Characteristics of particular patients with IH are presented in Table 1. None of the patients had positive thyroid antibodies (0 out of 6 recorded). Vitamin D deficiency (<30 ng/mL) was found in all patients with IH (6 out of 6 recorded), whereas severe Vitamin D deficiency (<20 ng/mL) was found in almost all patients but one (5 out of 6 recorded). Insulin resistance index (IRI) was increased (>1.25) in five out of six patients recorded. Either overweight (BMI ≥ 25 kg/m^2^) or obesity (BMI ≥ 30 kg/m^2^) was observed in four out of seven patients recorded.

When comparing mean values of different linear parameters between patients with IH and patients with normal thyroid tests (also after excluding individuals on L-thyroxine treatment) (Table 2), an obviously significantly lower FT4 concentration was found in the former group. Mean concentration of Vitamin D was lower in females with IH versus patients with normal thyroid tests (also after excluding individuals on L-thyroxine treatment); borderline significance was found when comparing to the whole population sample (also after excluding individuals on L-thyroxine treatment). The mean value of IRI was significantly higher in patients with IH compared to all other groups considered. When we applied FDR correction, vitamin D concentration and IRI unfortunately lost this statistical significance, and FT4 concentration expectedly remained statistically significant.

No statistically significant differences were found concerning any other measured linear parameters (Table 2).

Regarding the percentage of abnormal lipid profile (Table 3), no statistically significant differences were found between patients with IH and other groups considered.

In turn, concerning positive thyroid antibodies, no statistically significant differences were found between patients with IH and other groups considered (Table 4). However, it should be stressed that in none of the patients with IH positive thyroid antibodies were observed, whereas in all other groups (without IH), the percentage of positive TPOAb or TgAb ranges from 10% to 21%.

The parameters associated (to a certain extent) with hypothyroxinemia (BMI as an index of overweight or obesity, Hgb and RBC as indirect indices of iron supply, platelets as a factor associated with pro- and antiangiogenic imbalance) differed between patients with IH and other groups considered (Table 5). Additionally, we added into this comparison two factors, i.e., vitamin D deficiency and the increased IRI, as our previous statistical analyses (Table 2) revealed potential association of these linear variables with IH.

Severe vitamin D deficiency (<20 ng/mL) was the only linear parameter that occurred more commonly in patients with IH (83% vs. 43–47%), but only with borderline significance (Table 5). However, when we compared (with the use of the Mann–Whitney rank sum test) median FT4 between patients with and without severe vitamin D deficiency in the group of patients with normal thyroid tests, we found a significantly lower median FT4 in patients with severe vitamin D deficiency, i.e., 1.17 vs. 1.24 (*p* = 0.033); in the group of patients with normal thyroid tests without LT4 treatment, this difference lost its statistical significance (1.16 vs. 1.21; *p* = 0.088).

Regarding IRI > 1.25, its prevalence was much higher in patients with IH (67% vs. 36–38%) but without statistical significance (Table 5).

When comparing mean FT4 concentration in patients with and without severe vitamin D deficiency, in the group with normal TSH (*n* = 288), a significantly lower FT4 concentration was found in vitamin D deficient subjects (Table 6A). In turn, FT4 concentration was significantly lower in patients with increased IRI, when the statistical evaluation was done in the group with normal TSH (also after excluding individuals on L-thyroxine treatment) (Table 6B).

When we checked possible correlations between FT4 concentration and all other linear parameters (Table 7), an obvious negative correlation with TSH concentration and a positive correlation with FT3 were found. Additionally, positive correlations were found with HDL cholesterol or the HDLC/cholesterol ratio and a negative correlation with LDL cholesterol or triglycerides.

Of great importance is the observation that FT4 concentration correlated positively with vitamin D concentration and negatively with the IRI value in the group of 288 inpatients with normal TSH (also after excluding individuals on L-thyroxine treatment) (Table 7).

Furthermore, a negative correlation was found between FT4 and BMI in patients with a normal TSH after excluding individuals on L-thyroxine treatment (Table 7).

Expectedly, FT4 concentration correlated positively with iron concentration in women with normal TSH (Table 7); however, it should be recalled that iron concentration was not recorded in patients with IH. When we applied FDR correction, all correlations in all groups retained statistical significance.

In the univariate regression analysis, we found that, among the measured linear variables, only the IRI value was statistically associated with IH (Table 8); other linear variables are not presented in the table. Therefore, on the basis of the univariate regression analysis, IRI should be treated as the only independent linear factor associated with IH.

## 4. Discussion

There is no single study estimating the prevalence of IH in women of childbearing age from the general population.

When the dataset of the third round of the National Health and Nutrition Examination Survey (NHANES III) performed between 1988 and 1994 among U.S. subjects was used, the prevalence of hypothyroxinemia in women of childbearing age was found to be approx. 4% [27]. However, this percentage is probably overestimated, as the population sample did also comprise a small percentage of pregnant individuals, was reduced by patients treated with L-thyroxine and, of great importance, total thyroxine concentration was the only thyroid test used to diagnose hypothyroxinemia [27], probably resulting in also counting patients with overt hypothyroidism.

In our population sample of 466 non-pregnant female inpatients of childbearing age, IH was found with the frequency of 1.72%. According to our knowledge, this is the first study estimating the prevalence of IH in women of reproductive age and this percentage seems to be reliable when comparing to the above cited American study [27]. However, the important limitation of our observation is the fact that we collected data of hospitalized female individuals, therefore our sample is not representative of the general population.

The main observation from the present retrospective analysis is that IH is significantly associated with severe vitamin D deficiency and with insulin resistance.

Whereas the association of vitamin D deficiency with thyroid autoimmune diseases and thyroid cancer was documented in clinical and experimental studies [28], its relationship with thyroid dysfunction is not clearly defined. However, published results suggest that vitamin D deficiency contributes to the decreased thyroid function. For example, the decreased vitamin D level was found in hypothyroid patients with Hashimoto thyroiditis of both sexes [29] or in female patients at preconception and postmenopausal age [30]. However, the relationship between vitamin D deficiency and thyroid hormone level after exclusion of the autoimmune process has not been confirmed in the literature.

In the present study, we found that almost all female patients at reproductive age with IH had severe vitamin D deficiency. Via what mechanism does vitamin D deficiency contribute to hypothyroxinaemia is unknown. The well documented immunomodulatory function of vitamin D and the contribution of vitamin D deficiency to autoimmune thyroid disease [31] should be neglected, as IH is repeatedly found in the literature to be not associated with thyroid antibodies [2]. Consistently, none of our patients with IH had positive thyroid antibodies. Some experimental evidence, such as a strong homology between the molecular structure of vitamin D and thyroid hormone receptors, the presence of a functional 1,25(OH)2D3 receptor in rat thyroid follicular cells, and inhibitory effects of 1,25(OH)2D3 on TSH action in FRTL-5 cells, recently reviewed [32], suggest the role of vitamin D in thyroid physiology. At the same time, evidence exists from clinical and experimental studies on the role of vitamin D in human fertility and, consistently, on the contribution of vitamin D deficiency to infertility via numerous mechanisms [33]. As severe vitamin D deficiency was, in our patients of childbearing age, significantly associated with IH, this type of mild thyroid hypofunction may be one of the mechanisms of unfavorable effects of vitamin D deficiency on fertility. Whether supplementation of patients with vitamin D deficiency and with IH reverses the decreased thyroid function is unknown and, therefore, requires further prospective longitudinal studies. Independently of how vitamin D affects thyroid function, it is generally accepted by different authorities that this micronutrient should be supplemented before and during pregnancy [34].

The next linear factor strongly associated with IH in the present analysis is insulin resistance index. There are already some published papers confirming such a relationship. In comparison to euthyroid pregnant patients, pregnant individuals with IH had higher insulin resistance and higher BMI; maternal FT4 was negatively associated with both these parameters [3]. In patients with diabetes mellitus type 2, lower free thyroid hormone levels were associated with high blood glucose and insulin resistance, which normalized with metabolic improvement of diabetes [35]. Consistently, we have observed in our analysis that IRI was higher in patients with IH versus individuals without IH, that IRI correlated negatively with FT4 and, of great importance, IRI appeared to be the only independent factor associated with IH in regression analysis. Whereas both abnormalities, i.e., hypothyroxinemia and insulin resistance, contribute unfavorably to reproduction, it is difficult to state which of these two is a causative factor. Results from one experimental study suggest that hypothyroxinemia does constitute this primary phenomenon; whereas exposure of pregnant mice to an antibacterial agent (Triclosan) resulted in both hypothyroxinemia and insulin resistance, treatment with L-thyroxine reversed the latter abnormality [36].

An unfavorable contribution of insulin resistance to reproduction has been confirmed in the literature. Insulin resistance, as a component of obesity and polycystic ovary syndrome, contributes to infertility [37]. Therapeutic options comprise dietary modification or medications, both of which improve insulin sensitivity [37,38]. It can be added that insulin resistance increases the risk of spontaneous abortion after assisted reproduction technology treatment [39,40].

Regardless of which of these two abnormalities, i.e., insulin resistance or IH, is a primary phenomenon, the former should be eliminated in women of childbearing age (but also in general population), leading to the improvement of reproductive health (and general health as well).

It is known that insulin resistance occurs commonly together with obesity and abnormal lipid profile. Concerning obesity, its association with IH was confirmed in different studies. For example, women with hypothyroxinemia had significantly increased BMI at preconception, in the first trimester and at the time of delivery [15]. In a recently published study comprising almost 35,000 participants, preconception overweight/obesity and first-trimester IH were independently associated with an increased risk of macrosomia; additionally, a synergistic effect of preconception overweight/obesity and IH during pregnancy was observed [41]. It is not known how obesity contributes to hypothyroxinemia. One of the mechanism could be insulin resistance, discussed above. The long-term effects of obesity are not limited to infertility and maternal health, but may also comprise certain abnormalities in the offspring [42,43].

Consistently, in the present study, BMI correlated negatively with FT4 concentration in patients with normal TSH after excluding individuals on L-thyroxine treatment. Additionally, in subjects with IH compared to those without IH, mean BMI tended to be higher and abnormal BMI tended to occur more frequently.

The association between abnormal lipid profile and IH, which we found in the present analysis, also requires some comments. Whereas an abnormal lipid profile was not observed more frequently in females with IH, HDL cholesterol correlated positively, whereas LDL cholesterol or triglycerides correlated negatively with FT4 concentration in different groups considered. Such associations are expected concerning the mechanism of thyroid hormones action on lipid profile, and similar results were found by Knight and coworkers in a pregnant population [3]. It is worth mentioning that similarly to the association between IH and abnormal lipid profile, observed in the present analysis, we found the association between high normal TSH and abnormal lipid profile in the same population sample [25], and also in our earlier prospective studies performed in non-pregnant women of reproductive age [44,45]. Therefore, it can be concluded that both IH and high normal TSH (which is associated with only a relative decrease of thyroxine concentration) contribute to the abnormal lipid profile in women of childbearing age.

Regarding other earlier documented risk factors for IH during pregnancy, mentioned in the Introduction section [2], the following should be commented on: iodine deficiency, iron deficiency, and age.

Concerning iodine deficiency, theoretically all women considered in the present retrospective analysis were iodine sufficient according to the fact that they are living in Poland—an area characterized currently by iodine sufficiency for the non-pregnant population [46,47]; however, urine iodine excretion was obviously not measured in this retrospective study. Therefore, iodine deficiency, as the main risk factor for hypothyroxinemia, will not be discussed.

Iron deficiency affects at least one third of the worldwide population, being most common in women of childbearing age, contributing substantially to pregnancy complications [48]. Iron deficiency was found to be strongly associated with hypothyroxinemia in women of childbearing age, in both the pregnant [49,50,51] and nonpregnant [49] populations. Therefore, to prevent iron deficiency in women of childbearing age is of great importance. Unfortunately, the contribution of iron deficiency to IH cannot be discussed with respect to the current results, as neither iron nor ferritin concentrations were recorded in the group with IH. However, a positive correlation between iron concentration and FT4 concentration in patients with normal thyroid tests, observed in the present study, confirms that iron may modify the level of thyroid hormone synthesis even if they are in acceptable reference ranges.

Whereas older age is mentioned in the literature as a risk factor for IH [23], we did not find any association between IH and age. To document any relationship between older age and IH in women at childbearing age probably requires a larger population sample.

Concerning autoimmune processes, no association between positive thyroid antibodies and IH was found in the present study, which is consistent with previously published results [2].

Independently of how strongly modified risk factors contribute to hypothyroxinemia and infertility, they should be, together with all other nutritional abnormalities, eliminated in women of childbearing age, especially in those planning pregnancy [52].

In the present study, we found that the following linear parameters are associated with IH in women of childbearing age: insulin resistance, increased BMI, abnormal lipid profile and vitamin D deficiency. The first three were earlier confirmed in pregnant women [2,23], and were therefore expected to also be confirmed in the non-pregnant population of childbearing age. In turn, the relationship between severe vitamin D deficiency and IH found in the present study in women of childbearing age is, according to our knowledge, the first observation published, and suggests that a similar evaluation should be performed during pregnancy.

However, it should be clearly stated that our patients did constitute a specific group of inpatients hospitalized for endocrine diseases. Therefore, our observations should be confirmed in non-hospitalized individuals from the general population.

Diagnosing IH is based on obtaining lower than normal concentrations of FT4. Therefore, it is only a biochemical finding. On the other hand, however, unfavorable pregnancy outcomes are observed in patients with IH suggesting that this biochemical change has clinical consequences. Regardless of the incompletely answered question “Is IH only a statistical issue or a real thyroid dysfunction?”, low FT4 concentration (below or even slightly above lower reference range) should always be of concern at preconception and, especially, during pregnancy.

Appropriate preconception management of thyroid dysfunction is crucial for successful fertilization and pregnancy outcome. Controversies exist in the literature regarding the management of IH. According to the most recent guidelines [1], IH during pregnancy should not be treated routinely. Whereas results from some studies do not confirm benefits resulting from L-thyroxine treatment, other authors present rationales for such pharmacological intervention [22,23,24]. A better understanding of the mechanisms of hypothyroxinemia will allow one to optimize decisions concerning whether to treat or not treat patients with IH. Additionally, in most patients with IH, L-thyroxine treatment is hopefully not required after eliminating the risk factors that actually caused this type of mild thyroid dysfunction.

Modified risk factors for IH, i.e., iodine deficiency, iron deficiency, obesity with its common component insulin resistance, environmental disruptors and, as documented in the present study, vitamin D deficiency, should be eliminated in all women of childbearing age, especially at preconception, as creating optimal conditions for the developing fetus should start before planning pregnancy. This kind of prevention is of great value not only to preserve normal thyroid function but also for general health.

## 5. Conclusions

Insulin resistance, increased BMI, and abnormal lipid profile are associated with isolated hypothyroxinemia in nonpregnant women of childbearing age, similarly to what has been observed in the pregnant population. Whereas the first two constitute potential causative factors, the abnormal lipid profile is a possible consequence of hypothyroxinemia. Additionally, severe vitamin D deficiency, found in the present study to be associated with isolated hypothyroxinemia, seems to be a new risk factor for this mild thyroid hypofunction, however this association requires further evaluation in larger population samples. As thyroid hormones affect all organs and tissues, reproductive organs included, hypothyroxinemia may unfavorably contribute to female fertility and generally to reproduction via direct mechanisms but also indirectly via different factors, including vitamin D deficiency, insulin resistance, obesity, and abnormal lipid profile, regardless of whether they are causative factors for IH or its consequence. To eliminate risk factors for hypothyroxinemia in women of childbearing age may improve reproductive and general health.

## Figures and Tables

**Table 1:** Characteristics of particular patients with isolated hypothyroxinemia. nr: not recorded.

No.	Age [years]	BMI [kg/m^2^]	Hgb [g/dL]	TSH [mIU/L]	FT4 [ng/dL]	FT3 [pg/mL]	TgAb [IU/mL]	TPOAb [IU/mL]	TSHRAb [IU/L]	Vit. D [ng/mL]	IRI	Main Diagnoses
1	15	27.4	12.3	1.69	0.84	2.91	11.22	10.46	0	22.8	1.52	hyperandrogenism overweight
2	19	19.9	11.6	1.59	0.92	2.95	64.31	8.26	1.14	17.3	1.59	oligomenorrhoea
3	17	23	13.5	1.78	0.8	3.08	13.05	8.28	0.41	9.3	0.99	secondary amenorrhoea
4	16	37.1	13.6	2.13	0.86	2.87	0	0	0.35	15.8	1.63	oligomenorrhoea, polycystic ovary syndrome, obesity
5	23	30.04	14.2	0.72	0.91	2.95	38.41	13.69	0	7.7	1.49	oligomenorrhoea, glucose intolerance, obesity
6	30	nr	13.6	1.7	0.87	3.28	0	13.34	0	nr	nr	oligomenorrhoea
7	36	28.7	14	2.8	0.89	2.64	nr	nr	nr	10	1.26	infertility, miscarriage (1), overweight
8	44	23	12.3	2	0.89	2.71	nr	nr	nr	nr	nr	depression

**Table 2:** Clinical/laboratory parameters in patients with isolated hypothyroxinemia (IH) vs. patients with normal thyroid tests (additionally after excluding individuals on L-thyroxine (LT4) treatment) and vs. the whole group of 466 inpatients minus patients with IH (additionally after excluding individuals on L-thyroxine (LT4) treatment).

	IH (*n* = 8)	Normal Thyroid Tests (*n* = 280)	Normal Thyroid Tests without LT4 Treatment (*n* = 240)	The Whole Group Minus IH Patients (*n* = 458)	The Whole Group Minus IH Patients without LT4 Treatment (*n* = 352)
Age [years]	25.00 ± 3.71 *n* = 8	29.03 ± 0.51 *n* = 280 *p* = 0.193	28.83 ± 0.55 *n* = 240 *p* = 0.215	29.39 ± 0.43 *n* = 458 *p* = 0.184	28.71 + 0.47 *n* = 352 *p* = 0.246
Body mass [kg]	72.01 ± 6.04 *n* = 8	71.60 ± 1.60 *n* = 199 *p* = 0.962	71.23 ± 1.12 *n* = 171 *p* = 0.9327	70.61 ± 1.21 *n* = 324 *p* = 0.867	69.04 + 1.37 *n* = 251 *p* = 0.720
Height [m]	163.14 ± 1.84 *n* = 8	165.8 ± 0.63 *n* = 198 *p* = 0.437	165.579 ± 0.71 *n* = 170 *p* = 0.454	165.69 ± 0.46 *n* = 325 *p* = 0.425	165.63 + 0.56 *n* = 252 *p* = 0.465
BMI [kg/m^2^]	27.02 ± 2.16 *n* = 7	25.94 ± 0.53 *n* = 208 *p* = 0.711	25.74 ± 0.56 *n* = 178 *p* = 0.653	25.58 ± 0.40 *n* = 334 *p* = 0.613	24.94 + 0.45 *n* = 257 *p* = 0.446
RBC [10^12^/L]	4.42 ± 0.11 *n* = 8	4.50 ± 0.02 *n* = 279 *p* = 0.576	4.51 ± 0.023 *n* = 239 *p* = 0.512	4.45 ± 0.02 *n* = 457 *p* = 0.843	4.46 + 0.02 *n* = 351 *p* = 0.794
Hgb [g/dL]	13.14 ± 0.33 *n* = 8	13.12 ± 0.05 *n* = 279 *p* = 0.965	13.15 ± 0.06 *n* = 239 *p* = 0.960	12.98 ± 0.04 *n* = 457 *p* = 0.658	13.01 + 0.05 *n* = 351 *p* = 0.726
WBC [10^9^/L]	7.95 ± 1.23 *n* = 8	7.09 ± 0.12 *n* = 278 *p* = 0.238	7.08 ± 0.13 *n* = 238 *p* = 0.232	6.92 ± 0.09 *n* = 456 *p* = 0.148	6.89 + 0.1 *n* = 350 *p* = 0.137
Neutrophils [10^9^/L]	4.57 ± 1.01 *n* = 8	3.76 ± 0.10 *n* = 278 *p* = 0.203	3.76 ± 0.11 *n* = 238 *p* = 0.204	3.67 ± 0.07 *n* = 456 *p* = 0.136	3.68 + 0.09 *n* = 350 *p* = 0.145
Lymphocytes [10^9^/L]	2.39 ± 0.22 *n* = 8	2.54 ± 0.04 *n* = 278 *p* = 0.551	2.53 ± 0.04 *n* = 238 *p* = 0.577	2.47 ± 0.03 *n* = 456 *p* = 0.754	2.45 + 0.04 *n* = 350 *p* = 0.815
Platelets [10^9^/L]	256.1 ± 15.27 *n* = 8	256.57 ± 3.40 *n* = 266 *p* = 0.997	256.65 ± 3.71 *n* = 228 *p* = 0.994	253.6 ± 2.88 *n* = 437 *p* = 0.893	252.56 + 3.18 *n* = 337 *p* = 0.850
FT4 [ng/dL]	0.87 ± 0.04 *n* = 8	1.22 ± 0.01 *n* = 280 *p* < 0.001	1.20 ± 0.01 *n* = 240 *p* < 0.001	1.29 ± 0.03 *n* = 437 *p* = 0.091	1.29 + 0.04 *n* = 351 *p* = 0.104
FT3 [pg/mL]	2.92 ± 0.07 *n* = 8	3.09 ± 0.02 *n* = 260 *p* = 0.155	3.12 ± 0.02 *n* = 222 *p* = 0.112	3.33 ± 0.14 *n* = 432 *p* = 0.689	3.39 + 0.16 *n* = 331 *p* = 0.657
TSH [mIU/L]	1.80 ± 0.21 *n* = 8	1.91 ± 0.05 *n* = 280 *p* = 0.738	1.90 ± 0.05 *n* = 240 *p* = 0.758	2.53 ± 0.31 *n* = 438 *p* = 0.757	2.05 + 0.14 *n* = 352 *p* = 0.788
TPOAb [IU/mL]	9.00 ± 2.04 *n* = 8	39.01 ± 5.68 *n* = 248 *p* = 0.413	30.74 ± 5.28 *n* = 211 *p* = 0.489	57.01 ± 5.95 *n* = 399 *p* = 0.329	37.74 + 5.09 *n* = 307 *p* = 0.432
TgAb [IU/mL]	21.16 ± 10.36 *n* = 6	57.73 ± 11.13 *n* = 241 *p* = 0.606	51.29 ± 12.48 *n* = 202 *p* = 0.679	93.15 ± 10.66 *n* = 391 *p* = 0.407	77.58 + 12.14 *n* = 295 *p* = 0.509
TSHRAb [IU/L]	0.32 ± 0.18 *n* = 6	0.28 ± 0.02 *n* = 234 *p* = 0.806	0.32 ± 0.03 *n* = 196 *p* = 0.760	1.09 ± 0.24 *n* = 379 *p* = 0.690	0.75 + 0.21 *n* = 287 *p* = 0.766
Cholesterol [mg/dL]	164.75 ± 7.78 *n* = 8	169.76 ± 1.87 *n* = 265 *p* = 0.645	168.96 ± 2.03 *n* = 227 *p* = 0.701	171.2 ± 1.68 *n* = 429 *p* = 0.605	169.28 + 1.88 *n* = 330 *p* = 0.710
HDLC [mg/dL]	51.62 ± 9.79 *n* = 8	54.77 ± 0.91 *n* = 265 *p* = 0.567	54.50 ± 0.95 *n* = 227 *p* = 0.591	55.59 ± 0.80 *n* = 429 *p* = 0.514	55.88 + 0.87 *n* = 330 *p* = 0.463
LDLC [mg/dL]	94.25.1 ± 9.23 *n* = 8	93.07 ± 1.75 *n* = 265 *p* = 0.908	92.99 ± 1.89 *n* = 227 *p* = 0.902	92.57 ± 1.44 *n* = 427 *p* = 0.875	91.14 + 1.59 *n* = 329 *p* = 0.763
HDLC/Cholesterol	0.31 ± 0.04 *n* = 8	0.33 ± 0.009 *n* = 264 *p* = 0.556	0.33 ± 0.006 *n* = 226 *p* = 0.547	0.33 ± 0.004 *n* = 427 *p* = 0.522	0.34 + 0.005 *n* = 328 *p* = 0.409
TGs [mg/dL]	107.5 ± 12.42 *n* = 8	98.55 ± 3.76 *n* = 264 *p* = 0.681	99.04 ± 4.28 *n* = 226 *p* = 0.712	98.5 ± 2.87 *n* = 427 *p* = 0.672	95.42 + 3.37 *n* = 329 *p* = 0.579
Glucose [mg/dL]	84.85 ± 2.55 *n* = 7	83.55 ± 0.64 *n* = 258 *p* = 0.740	83.21 ± 0.60 *n* = 220 *p* = 0.630	84.68 ± 1.06 *n* = 418 *p* = 0.983	84.54 + 1.32 *n* = 323 *p* = 0.972
CRP	0.66 ± 0.40 *n* = 4	0.34 ± 0.07 *n* = 92 *p* = 0.366	0.33 ± 0.08 *n* = 80 *p* = 0.371	0.69 ± 0.13 *n* = 186 *p* = 0.970	0.77 ± 0.187 *n* = 132 *p* = 0.916
Vit D [ng/mL]	13.82 ± 2.37 *n* = 6	20.83 ± 0.56 *n* = 223 *p* = 0.044	20.61 ± 0.60 *n* = 175 *p* = 0.044	21.57 ± 0.51 *n* = 375 *p* = 0.057	21.50 + 0.59 *n* = 276 *p* = 0.059
IRI	1.41 ± 0.01 *n* = 6	1.10 ± 0.02 *n* = 150 *p* = 0.033	1.12 ± 0.03 *n* = 132 *p* = 0.043	1.1 ± 0.02 *n* = 222 *p* = 0.036	1.09 + 0.02 *n* = 183 *p* = 0.037

Comparison between subgroups was performed by Student’s unpaired *t*-test. Statistical significance was determined at the level of *p* < 0.05. Statistically significant differences are shaded.

**Table 3:** Percentage of abnormal lipid profile in patients with isolated hypothyroxinemia (IH) vs. patients with normal thyroid tests (additionally after excluding individuals on L-thyroxine (LT4) treatment) and vs. the whole group of 466 inpatients minus patients with IH (additionally after excluding individuals on L-thyroxine (LT4) treatment).

	IH (*n* = 8)	Normal Thyroid Tests (*n* = 265)	Normal Thyroid Tests without LT4 Treatment (*n* = 227)	The Whole Group Minus IH Patients (*n* = 429)	The Whole Group Minus IH Patients without LT4 Treatment (*n* = 330)
Cholesterol ≥200 mg/dL	*n* = 0 0%	*n* = 39 15% *p* = 0.237	*n* = 32 14% *p* = 0.253	*n* = 82 19% *p* = 0.172	*n* = 56 17% *p* = 0.202
HDLC <40 mg/dL	*n* = 2 25%	*n* = 36 14% *p* = 0.382	*n* = 30 13% *p* = 0.328	*n* = 65 15% *p* = 0.435	*n* = 43 13% *p* = 0.323
LDLC >100 mg/dL	*n* = 2 25%	*n* = 98 37% *p* = 0.488	*n* = 84 37% *p* = 0.487	*n* = 156 36% *p* = 0.521	*n* = 117 35% *p* = 0.557
TGs >150 mg/dL	*n* = 1 12%	*n* = 25 9% *p* = 0.771	*n* = 23 10% *p* = 0.853	*n* = 54 13% *p* = 0.934	*n* = 32 10% *p* = 0.853
HDLC/cholesterol <0.2	*n* = 1 12%	*n* = 20 8% *p* = 0.683	*n* = 15 7% *p* = 0.590	*n* = 35 8% *p* = 0.681	*n* = 21 6% *p* = 0.485

Statistical evaluation was performed by the two-sided ratio comparison test. Statistical significance was determined at the level of *p* < 0.05.

**Table 4:** Percentage of positive thyroid antibodies in patients with isolated hypothyroxinemia (IH) vs. patients with normal thyroid tests (additionally after excluding individuals on L-thyroxine (LT4) treatment) and vs. the whole group of 466 inpatients minus patients with IH (additionally after excluding individuals on L-thyroxine (LT4) treatment).

	IH *n* = 6	Normal Thyroid Tests *n* = 228	Normal Thyroid Tests without LT4 Treatment *n* = 183	The Whole Group Minus IH Patients *n* = 403	The Whole Group Minus IH Patients without LT4 Treatment (*n* = 307)
TPOAb ≥34 IU/mL	*n* = 0 0%	*n* = 32 14% *p* = 0.325	*n* = 21 11% *p* = 0.391	*n* = 83 21% *p* = 0.208	*n* = 43 14% *p* = 0.324
TgAb ≥115 IU/mL	*n* = 0 0%	*n* = 27 11% *p* = 0.391	*n* = 18 10% *p* = 0.416	*n* = 76 19% *p* = 0.237	*n* = 44 14% *p* = 0.324
TSHRAb ≥1.75 IU/mL	*n* = 0 0%	*n* = 1 0.4% *p* = 0.877	*n* = 1 0.5% *p* = 0.575	*n* = 18 4% *p* = 0.671	*n* = 9 3% *p* = 0.667

Statistical evaluation was performed by the two-sided ratio comparison test. Statistical significance was determined at the level of *p* < 0.05.

**Table 5:** Percentage of abnormal values of chosen parameters potentially associated with hypothyroxinemia in patients with isolated hypothyroxinemia (IH) vs. patients with normal thyroid tests (additionally after excluding individuals on L-thyroxine (LT4) treatment) and vs. the whole group of 466 inpatients minus patients with IH (additionally after excluding individuals on L-thyroxine (LT4) treatment).

	IH	Normal Thyroid Tests	Normal Thyroid Tests without LT4 Treatment	The Whole Group Minus IH Patients	The Whole Group Minus IH Patients without LT4 Treatment
BMI > 25 [kg/m^2^]	*n* = 4 out of 7 57%	*n* = 83 out of 208 40% *p* = 0.369	*n* = 67 out of 171 39% *p* = 0.341	*n* = 129 out of 341 38% *p* = 0.307	*n* = 89 out of 257 35% *p* = 0.231
Hgb <12/>15 [g/dL]	*n* = 1 out of 8 12%	*n* = 37 out of 279 13% *p* = 0.276	*n* = 32 out of 227 14% *p* = 0.256	*n* = 81 out of 465 17% *p* = 0.708	*n* = 63 out of 351 18% *p* = 0.662
RBC <3.8/>5.8 [10^12^/L]	*n* = 0 out of 8 0%	*n* = 7 out of 279 2% *p* = 0.689	*n* = 5 out of 227 2% *p* = 0.517	*n* = 22 out of 465 5% *p* = 0.571	*n* = 16 out of 351 5% *p* = 0.517
Platelets <150/>400 [10^9^/L]	*n* = 0 out of 8 0%	*n* = 6 out of 265 2% *p* = 0.687	*n* = 4 out of 227 2% *p* = 0.686	*n* = 20 out of 465 4% *p* = 0.564	*n* = 14 out of 351 4% *p* = 0.564
Vit D < 30 [ng/mL]	*n* = 6 out of 6 100%	*n* = 192 out of 223 86% *p* = 0.312	*n* = 152 out of 175 87% *p* = 0.385	*n* = 310 out of 375 83% *p* = 0.298	*n* = 230 out of 276 83% *p* = 0.299
Vit D < 20 [ng/mL]	*n* = 5 out of 6 83%	*n* = 101 out of 223 45% *p* = 0.053	*n* = 83 out of 175 47% *p* = 0.084	*n* = 163 out of 375 43% *p* = 0.051	*n* = 123 out of 276 44% *p* = 0.054
IRI > 1.25	*n* = 4 out of 6 67%	*n* = 54 out of 150 36% *p* = 0.125	*n* = 49 out of 129 38% *p* = 0.157	*n* = 79 out of 222 36% *p* = 0.128	*n* = 67 out of 183 37% *p* = 0.137

Statistical evaluation was performed by the two-sided ratio comparison test. Statistical significance was determined at the level of *p* < 0.05.

**Table 6:** Mean FT4 concentration in patients with/without severe vitamin D deficiency (<20 ng/mL/≥20 ng/mL) (**A**) or with/without abnormal IRI (>1.25/≤1.25) (**B**), evaluated in the group of 288 inpatients with normal TSH (and also after excluding individuals on LT4 treatment).

**A**	**FT4**		***p***
	**Vit. D < 20 [ng/mL]**	**Vit. D > 20 [ng/mL]**	
Group of 288 patients with normal TSH	1.193 ± 0.018 *n* = 106	*1.240* ± 0.014 *n* = 123	0.042
Group of 288 patients with normal TSH without LT4 treatment	1.176 ± 0.018 *n* = 92	1.215 ± 0.015 *n* = 99	0.101
**B**	**FT4**		***p***
	**IRI** **>1.25**	**IRI** **<1.25**	
Group of 288 patients with normal TSH	*1.153* ± 0.022 *n* = 59	1.225 ± 0.017 *n* = 97	0.009
Group of 288 patients with normal TSH without LT4 treatment	1.131 ± 0.019 *n* = 55	1.221 ± 0.018 *n* = 83	0.001

Comparison between subgroups was performed by Student’s unpaired *t*-test. Statistical significance was determined at the level of *p* < 0.05. Statistically significant differences are shaded.

**Table 7:** Correlations between FT4 concentration and clinical/laboratory parameters in the four groups considered. Statistical evaluation was performed by the Pearson’s correlation test. Statistical significance was determined at the level of *p* < 0.05.

	FT4 Concentration			
	Group of 288 Patients with Normal TSH	Group of 288 Patients with Normal TSH without LT4 Treatment	The Whole Group of 466 Patients	The Whole Group of Patients without LT4 Treatment
Age [years]	r = 0.069 *p* = 0.244 *n* = 288	r = 0.029 *p* = 0.644 *n* = 248	r = 0.094 *p* = 0.043 *n* = 465	r = 0.106 *p* = 0.044 *n* = 359
Body mass [kg]	r = −0.081 *p* = 0.248 *n* = 206	r = −0.149 *p* = 0.046 *n* = 178	r = −0.033 *p* = 0.553 *n* = 331	r = −0.065 *p* = 0.301 *n* = 257
Height [m]	r = −0.170 *p* = 0.01 *n* = 205	r = −0.158 *p* = 0.036 *n* = 177	r = −0.016 *p* = 0.770 *n* = 332	r = −0.014 *p* = 0.855 *n* = 258
BMI [kg/m^2^]	r = −0.110 *p* = 0.107 *n* = 215	r = −0.206 *p* = 0.004 *n* = 185	r = −0.041 *p* = 0.448 *n* = 340	r = −0.087 *p* = 0.158 *n* = 263
RBC [10^12^/L]	r = 0.058 *p* = 0.322 *n* = 287	r = 0.036 *p* = 0.576 *n* = 247	r = 0.044 *p* = 0.347 *n* = 464	r = 0.006 *p* = 0.904 *n* = 358
Hgb [g/dL]	r = 0.084 *p* = 0.154 *n* = 287	r = 0.049 *p* = 0.441 *n* = 247	r = −0.073 *p* = 0.116 *n* = 464	r = −0.099 *p* = 0.061 *n* = 358
WBC [10^9^/L]	r = −0.007 *p* = 0.904 *n* = 286	r = −0.070 *p* = 0.273 *n* = 246	r = 0.011 *p* = 0.818 *n* = 463	r = 0.034 *p* = 0.517 *n* = 357
Neutrophils [10^9^/L]	r = 0.018 *p* = 0.758 *n* = 286	r = −0.028 *p* = 0.660 *n* = 246	r = 0.067 *p* = 0.148 *n* = 463	r = 0.092 *p* = 0.080 *n* = 357
Lymphocytes [10^9^/L]	r = −0.048 *p* = 0.414 *n* = 286	r = −0.098 *p* = 0.123 *n* = 246	r = −0.140 *p* = 0.002 *n* = 463	r = −0.128 *p* = 0.015 *n* = 357
Platelets [10^9^/L]	r = −0.006 *p* = 0.920 *n* = 273	r = −0.037 *p* = 0.574 *n* = 236	r = 0.133 *p* = 0.005 *n* = 444	r = 0.143 *p* = 0.008 *n* = 344
TSH [mIU/L]	r = −0.141 *p* = 0.017 *n* = 288	r = −0.155 *p* = 0.014 *n* = 248	r = −0.176 *p* < 0.001 *n* = 465	r = −0.187 *p* < 0.001 *n* = 359
FT3 [pg/mL]	r = 0.296 *p* = 0.000 *n* = 268	r = 0.431 *p* < 0.001 *n* = 230	r = 0.922 *p* < 0.001 *n* = 439	r = 0.928 *p* < 0.001 *n* = 338
TPOAb [IU/mL]	r = 0.111 *p* = 0.077 *n* = 254	r = 0.056 *p* = 0.408 *n* = 217	r = 0.118 *p* = 0.017 *n* = 406	r = 0.173 *p* = 0.002 *n* = 312
TgAb [IU/mL]	r = 0.037 *p* = 0.560 *n* = 246	r = −0.007 *p* = 0.918 *n* = 208	r = 0.082 *p* = 0.104 *n* = 396	r = 0.138 *p* = 0.017 *n* = 300
TSHRAb [IU/L]	r = 0.083 *p* = 0.199 *n* = 238	r = 0.055 *p* = 0.434 *n* = 202	r = 0.592 *p* < 0.001 *n* = 385	r = 0.863 *p* < 0.001 *n* = 293
Cholesterol [mg/dL]	r = −0.013 *p* = 0.832 *n* = 273	r = −0.063 *p* = 0.332 *n* = 235	r = −0.261 *p* < 0.001 *n* = 436	r = −0.261 *p* < 0.001 *n* = 337
HDLC [mg/dL]	r = 0.113 *p* = 0.063 *n* = 411	r = 0.132 *p* = 0.043 *n* = 235	r = −0.084 *p* = 0.077 *n* = 436	r = −0.095 *p* = 0.082 *n* = 337
LDLC [mg/dL]	r = −0.223 *p* = 0.062 *n* = 273	r = −0.103 *p* = 0.117 *n* = 235	r = −0.221 *p* < 0.001 *n* = 434	r = −0.226 *p* < 0.001 *n* = 336
HDLC/Cholesterol	r = 0.133 *p* = 0.028 *n* = 272	r = 0.178 *p* = 0.006 *n* = 234	r = 0.117 *p* = 0.014 *n* = 434	r = 0.136 *p* = 0.013 *n* = 335
TGs [mg/dL]	r = −0.102 *p* = 0.093 *n* = 272	r = −0.142 *p* = 0.03 *n* = 234	r = −0.083 *p* = 0.085 *n* = 434	r = −0.092 *p* = 0.093 *n* = 336
Glucose [mg/dL]	r = 0.058 *p* = 0.348 *n* = 265	r = −0.073 *p* = 0.269 *n* = 227	r = 0.103 *p* = 0.034 *n* = 424	r = 0.098 *p* = 0.074 *n* = 329
Fe [µg/dL]	r = 0.392 *p* = 0.016 *n* = 37	r = 0.239 *p* = 0.196 *n* = 31	r = −0.162 *p* = 0.173 *n* = 70	r = −0.160 *p* = 0.279 *n* = 54
Vit D [ng/mL]	r = 0.154 *p* = 0.020 *n* = 229	r = 0.153 *p* = 0.035 *n* = 191	r = 0.007 *p* = 0.882 *n* = 380	r = −0.001 *p* = 0.982 *n* = 281
IRI	r = −0.178 *p* = 0.026 *n* = 156	r = −0.241 *p* = 0.004 *n* = 138	r = −0.036 *p* = 0.584 *n* = 227	r = −0.093 *p* = 0.202 *n* = 188

Statistically significant differences are shaded. R—Pearson’s correlation coefficient.

**Table 8:** Univariate logistic regression analysis of isolated hypothyroxinemia (IH) determinants performed in specified groups. Results are presented only for insulin resistance index (IRI).

Chosen Group	Univariate Regression		
	OR	95%Cl	*p*
Group of 288 patients with normal TSH	25.57	0.06–6.42	0.046
Group of 288 patients with normal TSH without LT4 treatment	3.79	−1.58–4.25	0.369
The whole group of 466 patients	19.12	0.082–5.82	0.044
The whole group of patients without LT4 treatment	30.51	0.521–6.31	0.021

Statistical significance was determined at the level of *p* < 0.05. Statistically significant differences are shaded.

## Data Availability

The datasets used and/or analyzed during the current study are available from the corresponding author on reasonable request.
